# Qualitative Treatment-Subgroup Interactions in a Randomized Clinical Trial of Treatments for Adolescents with ADHD: Exploring What Cognitive-Behavioral Treatment Works for Whom

**DOI:** 10.1371/journal.pone.0150698

**Published:** 2016-03-15

**Authors:** Bianca E. Boyer, Lisa L. Doove, Hilde M. Geurts, Pier J. M. Prins, Iven Van Mechelen, Saskia Van der Oord

**Affiliations:** 1 Clinical Psychology, KU Leuven, Leuven, Belgium; 2 Department of Developmental Psychology, University of Amsterdam, Amsterdam, The Netherlands; 3 Centre for Cognitive Science, University of Amsterdam, Amsterdam, The Netherlands; 4 Dutch Autism & ADHD research center (d’Arc), Department of Brain and Cognition, University of Amsterdam, Amsterdam, The Netherlands; 5 Research Group of Quantitative Psychology and Individual Differences, KU Leuven, Leuven, Belgium; Erasmus University Rotterdam, NETHERLANDS

## Abstract

**Objective:**

This study explored qualitative treatment-subgroup interactions within data of a RCT with two cognitive behavioral treatments (CBT) for adolescents with ADHD: a planning-focused (PML) and a solution-focused CBT (SFT). Qualitative interactions imply that which treatment is best differs across subgroups of patients, and are therefore most relevant for personalized medicine.

**Methods:**

Adolescents with ADHD (*N* = 159) received either PML or SFT. Pre-, post- and three-month follow-up data were gathered on parent-rated ADHD symptoms and planning problems. Pretreatment characteristics were explored as potential qualitative moderators of pretest to follow-up treatment effects, using an innovative analyses technique (QUINT; Dusseldorp & Van Mechelen, 2014). In addition, qualitative treatment-subgroup interactions for the therapeutic changes from pre- to posttest and from post- to follow-up test were investigated.

**Results:**

For the entire time span from pretest to follow-up only a quantitative interaction was found, while from posttest to follow-up qualitative interactions were found: Adolescents with less depressive symptoms but more anxiety symptoms showed more improvement when receiving PML than SFT, while for other adolescents the effects of PML and SFT were comparable.

**Discussion:**

Whereas subgroups in both treatments followed different trajectories, no subgroup was found for which SFT outperformed PML in terms of the global change in symptoms from pretest to three months after treatment. This implies that, based on this exploratory study, there is no need for personalized treatment allocation with regard to the CBTs under study for adolescents with ADHD. However, for a subgroup with comorbid anxiety symptoms but low depression PML clearly appears the treatment of preference.

**Trial Registration:**

Nederlands Trial Register NTR2142

## Introduction

Attention Deficit Hyperactivity Disorder (ADHD) is a childhood neuro-developmental disorder that affects approximately 5–10% of the children [1] [2]. Children with the disorder have symptoms of inattention, symptoms of hyperactivity/impulsivity or both, resulting in three subtypes of the disorder [3] [4]: the inattentive subtype, the hyperactive/impulsive subtype and the combined subtype. The trajectories of these symptom-clusters differ, as symptoms of inattention usually persist during development, while symptoms of hyperactivity/impulsivity often diminish over time. About 65% of children with ADHD still meet criteria for the diagnosis in adolescence [5] [6] In addition, about 62% of children with ADHD below 19 years have at least one comorbid disorder, whereas 34% even have two or more comorbid conditions [7]. Prevalent comorbid disorders are oppositional defiant disorder (ODD), conduct disorder (CD), anxiety- and mood-disorders, adjustment disorder and substance use disorder [7]. Also, more boys than girls have ADHD (3:1 respectively [8]) and girls appear to have less severe inattention, hyperactivity and impulsivity, but greater intellectual impairments than boys with ADHD [8]. In addition, girls tend to have more internalizing comorbid disorders than boys, whilst boys with ADHD are at higher risk for externalizing psychiatric comorbidities than girls [7]. Taken together, the group of individuals with ADHD is a heterogeneous one.

This heterogeneity makes it unlikely for one treatment to fit all and increases the need for personalized treatment. Randomized clinical trials (RCTs) can be valuable in revealing moderators of therapeutic change, investigating for whom what treatment is most effective [9]. For example, in a recent RCT the effectiveness of two cognitive behavioral treatments (CBTs) for adolescents with ADHD was investigated [10]. Even though some have been critical of the use of CBT in children with ADHD [11], others presume that adolescents might have enough cognitive capacity to benefit from CBT [12]. Because adolescents with ADHD have planning problems in daily life that can cause impairment in school, family- and social functioning [13] and evidence-based non-pharmacological treatments for adolescents with ADHD are lacking [14], a CBT was developed focusing on planning skills: Plan My Life (PML) [15]. In PML, every session a fixed, planning skills focused, subject and strategy is discussed and trained (e.g., a to-do list). In a multi-site RCT (*N* = 16 sites, *N* = 56 therapists [10]), this treatment was compared to a control CBT, without the proposed active element of enhancing planning skills: a solution-focused treatment (SFT [16]). Both PML and SFT are individual, manualized treatments consisting of 8 adolescent sessions and 2 parental sessions. Whereas in PML every week planning skills are actively learned by discussing a fixed subject, in SFT the adolescent/parent chooses a problem that is discussed using fixed questions in a solution focused manner, to lead the adolescent to a solution for the problem. To reduce drop-out, motivational interviewing is integrated within both treatments.

Pre-, post- and three-month follow-up data were gathered in 159 adolescents with ADHD (12 to 17 years), with parent-rated ADHD symptoms and planning problems as primary outcomes. Results showed a significant improvement of primary outcomes as well as comorbid symptoms, functioning and impairment (with large effect sizes) from pre- to posttest with maintenance of effects to three months after treatment on most measures, also when controlling for medication use. In addition, 15.2% of adolescents showed normalization of functioning at follow-up. However, only marginally significant treatment differences were found, in favor of PML: PML showed more reduction of parent-rated planning problems compared to SFT, and higher treatment satisfaction of parents and therapists [10]. Also, due to the lack of an adequate control-group like a waitlist or a treatment as usual group, efficacy of both treatments could not be proven. The lack of differences in treatment outcome in this RCT could be due to heterogeneity of treatment effects in different subgroups of adolescents with ADHD: if for specific subgroups of adolescents one treatment is better than the other, while for other subgroups the reverse is true, this can result in comparable mean outcomes for the two treatments.

This phenomenon, where the optimal treatment for one subgroup differs from that for another subgroup, is referred to as a *qualitative* treatment-subgroup interaction, as opposed to a *quantitative* treatment-subgroup interaction, where the optimal treatment is the same in all subgroups but the size of the between-treatment difference differs across subgroups [17] [18]. In both cases, the patient characteristic(s) defining the subgroups in question are called moderators of treatment effect [9]. For example, Ogrodniczuk et al. [19] compared two forms of short-term individual psychotherapy (interpretative, supportive) for individuals suffering from depression. The authors found that for males interpretative therapy outperforms supportive therapy, whereas for females the reverse is true. This is a classic example of a *qualitative* treatment-subgroup interaction, where gender acts as a moderator of treatment effect. In the case of a *quantitative* treatment-subgroup interaction, the same treatment, for example interpretative therapy, would have outperformed supportive therapy in both genders, but for males the difference between the two treatments would have been larger than for females.

In the present study, a new and innovative statistical technique is used to explore these *qualitative* interactions: QUalitative INteraction Trees (QUINT [18]) (note that the meaning of the term “qualitative” as used in the context of qualitative treatment-subgroup interactions should not be confused with the meaning of the same term in the context of qualitative research methods). Earlier work on the detection of treatment-subgroup interactions primarily refers to situations in which clear a priori hypotheses exist about which subgroups of clients are involved in the interactions, or situations that involve a small number of potential moderator variables only. Examples include factorial analyses of variance (ANOVA), with one factor pertaining to treatment methods and another one to subgroups [20], and regression analyses with suitable interaction terms being included in the regression model (see e.g., [21] [22]). In contrast, QUINT does not require a priori hypotheses or a limited number of potential moderator variables but rather induces subgroups involved in treatment-subgroup interactions during the actual data analysis. QUINT further differs from other methods that induce subgroups during the actual data analysis (for a review, see [23]) in that it focuses on subgroups involved in clinically significant qualitative treatment-subgroup interactions; these are of particular relevance for personalized treatment assignment. The goal of QUINT is to find a partition of the total group of adolescents with ADHD, based on their pretreatment characteristics, into two or three mutually exclusive types of subgroups that are characterized as follows: In the first subgroup type PML outperforms SFT; in the second subgroup type the reverse is true; and in the third (optional) subgroup type, the adolescents assigned to PML would show more or less the same outcome as the adolescents assigned to SFT. Each subgroup type may comprise one or several subgroups of adolescents as defined by a combination of one or several dichotomized patient pretreatment characteristics (for which associated cut-off scores are being provided by QUINT). For example, a combination of pretreatment scores for intellectual impairment and externalization larger than some critical values may imply that SFT yields better results than PML, whereas for other subgroups the reverse holds true. As such, the QUINT results may have straightforward implications for optimal treatment assignment strategies to support healthcare decision makers of adolescents with ADHD. Note, however, that the results of a QUINT analysis may also be that the total group of clients is not partitioned, that is, that no subgroups involved in a qualitative treatment-subgroup interaction can be identified. For an example of a recent successful application of QUINT in the field of psychotherapy research, see [24].

The present study is the first to investigate *qualitative* interactions in treatments for adolescents with ADHD, comparing two types of CBT. To our knowledge, there are no studies on qualitative interactions in the treatment of ADHD and this is the first study using QUINT to investigate this. Similar to other tree-based methods, QUINT relies on a large search space based on a large number of covariate split-point combinations and therefore needs large sample sizes [18]. Even though this study is the largest RCT in adolescents with ADHD to date, for QUINT to show replicable results a larger sample size would be preferred. Dusseldorp and Van Mechelen [18] state that the safest way to deal with QUINT is as an exploratory tool to generate meaningful hypotheses that should be tested in follow-up confirmatory research with new RCTs. This RCTs should make use of a stratified sampling scheme in which the strata are constructed on the basis of the splitting variables and split points as identified by QUINT [18]. This is especially the case in applications with relatively smaller sample sizes. Therefore, this study should be considered exploratory, aimed at generating hypotheses for further research on treatment allocation in adolescents with ADHD.

To date, no treatment moderation studies have been conducted in adolescents with ADHD. However, two research groups *have* investigated a wide range of treatment moderators (child and family characteristics) in younger children with ADHD (up to 12 years of age), using mainly traditional moderation analyses such as ANOVA in which clear a priori hypotheses about potential moderators are required] [25] [26] [27] [28] [29] [30] [31] [32]. Although solely based on behavioral (parent) treatment of children with ADHD as compared to other types of treatment (i.e., medication, combined treatment or regular care), the following characteristics appeared to positively influence the effects of behavioral (parent) treatment on ADHD-symptoms: having no or one single comorbid disorder [30], in particular anxiety [25] [27], being older of age, having a mother with high parenting self-efficacy [30], or having no or one single DAT1 10-repeat allele [31]. Due to a lack of treatment moderation studies in adolescents with ADHD, and taking the heterogeneity of our sample into consideration, we measured a broad range of pretreatment patient characteristics to explore as potentially relevant moderator variables in the present study: age, gender, full-scale IQ, medication use, parental education, ADHD subtype, ADHD severity, comorbid ODD/CD-, depressive- and anxiety symptoms, and overall impairment. In addition, planning problems were included as a potential moderator, as one treatment aimed at enhancing planning skills (PML), whereas the other treatment did not (SFT). Inclusion of moderators in this study was based on1) moderators in previous research (e.g., gender, medication use, IQ, age, parental education, comorbid internalizing disorders like depressive and/or anxiety, externalizing disorders like ODD and/or CD), and 2) because one can hypothesize one CBT to be a better fit than the other. For example, even though this has never been investigated one could argue that older adolescents, adolescents with higher IQs, or less ADHD symptoms or impairment or adolescents with parents who have higher education, have more cognitive capacity or support at home, and therefore do better in a more open treatment like SFT, while younger adolescents, or with lower IQs, more severe ADHD or impairment and less educated parents fare better with a more structured treatment like PML. Outcome measures in this study were the primary outcomes from our RCT: parent-rated ADHD-symptoms and planning problems [10].

To our knowledge, previous studies on moderation of treatment effects in children with ADHD have focused mainly on moderators of short-term effects of treatment between pre- and posttest ([25] [26] [27] [28] [29] [30] [31] [32]; with the exception of [33]). However, trajectories of treatment effects may differ between phases of treatment. The best-known example thereof is the MTA-study, where children who had received medication only or in combination with behavior therapy improved more from pre- to posttest than children who had received behavior therapy only or community care [34]. However, 10 months after treatment these differences had diminished and approximately 2 years after treatment, groups did not differ significantly on any measure, an effect that remained stable until 8 years after pretest [33] [35] [36]. Also, even though in the MTA-study moderators of treatment effect were found from pre- to posttest [25] [26] [27] [28] [29], no moderators of treatment effect were found from pretest to 36 months after treatment [33].

Therefore, in the present study, apart from investigating qualitative moderators of treatment effects from pretest to follow-up, also qualitative moderators of the immediate treatment effect (from pretest to posttest) and of treatment effect maintenance (from posttest to follow-up three months after treatment) will be examined. More specifically, using QUINT, we investigated the following primary research question: Are qualitative treatment–subgroup interactions present in the data of our RCT (from pretest to three months after treatment)? And, if so, which treatment is best for which subgroup of adolescents with ADHD? A secondary research question we investigated, was whether there are qualitative treatment-subgroup interactions in the two phases of the trajectory of therapeutic change from pretest to follow-up, namely from pretest to posttest, and from posttest to follow-up? Given the lack of prior research on moderators of treatment effects in adolescents with ADHD, and in particular on moderators involved in qualitative treatment-subgroup interactions, we stated no specific expectations. When indeed qualitative treatment-subgroup interactions would be found from pretest to follow-up, this will generate hypothesis that can be tested in a follow-up RCT. This would be highly relevant for personalized medicine, as it would enable allocation of adolescents with ADHD to specific treatments, resulting in higher treatment efficacy.

## Methods

For a more detailed description of the sample, treatment content, procedures, research design, and approach to missing data, we refer to Boyer, Geurts, Prins, and Van der Oord [10].

### Trial design

This was a multi-center (16 sites), two-arm parallel-group randomized clinical trial conducted in the Netherlands (Clinical trials registration “Training adolescents with ADHD to plan and organize: Investigating short- and longterm effects of treatment”, http://www.trialregister.nl/trialreg/admin/rctview.asp?TC=2142, NTR2142). No important changes to methods were made after trial commencement. The date range for participant recruitment was September 2010 until October 2012, the date range for three-month follow-up was February 2011 until April 2013. The CONSORT checklist, original trial study protocol, translated trial study protocol, original ethics protocol, and translated ethics protocol are available as CONSORT Checklist in S1 CONSORT Checklist, Original Protocol in S1 Protocol, Translated Protocol in S2 Protocol, Original Ethics Protocol in S3 Protocol, and Translated Ethics Protocol in S4 Protocol.

### Participants and procedure

All participants (*N* = 159) were adolescents aged 12 to 17 years (*M*_age_ = 14.4 years), who attended secondary school. Participants had received a prior DSM-IV-TR diagnosis of ADHD [3] by a child psychiatrist or certified psychologist, which was confirmed with the Diagnostic Interview Schedule for Children for DSM-IV parent version (DISC-IV [37]). The DISC-IV is a structured diagnostic interview based on DSM-IV, which establishes ADHD group membership based on a diagnostic algorithm, including a check for the presence of cross-situational impairment. Participants had a full scale IQ (FSIQ) > 80 measured by the short version of the Dutch Wechsler Intelligence Scale for Children (WISC-III [38] [39]). During treatment 124 adolescents used psychostimulants (no atomoxetine) and were requested to keep the dose stable until posttest. Note that after posttest adolescents could change their medication use or dose. Exclusion criteria included having a comorbid autism spectrum disorder, depression with suicidal ideations, acute familial crisis, CD or predominant addiction.

**Fig 1:**
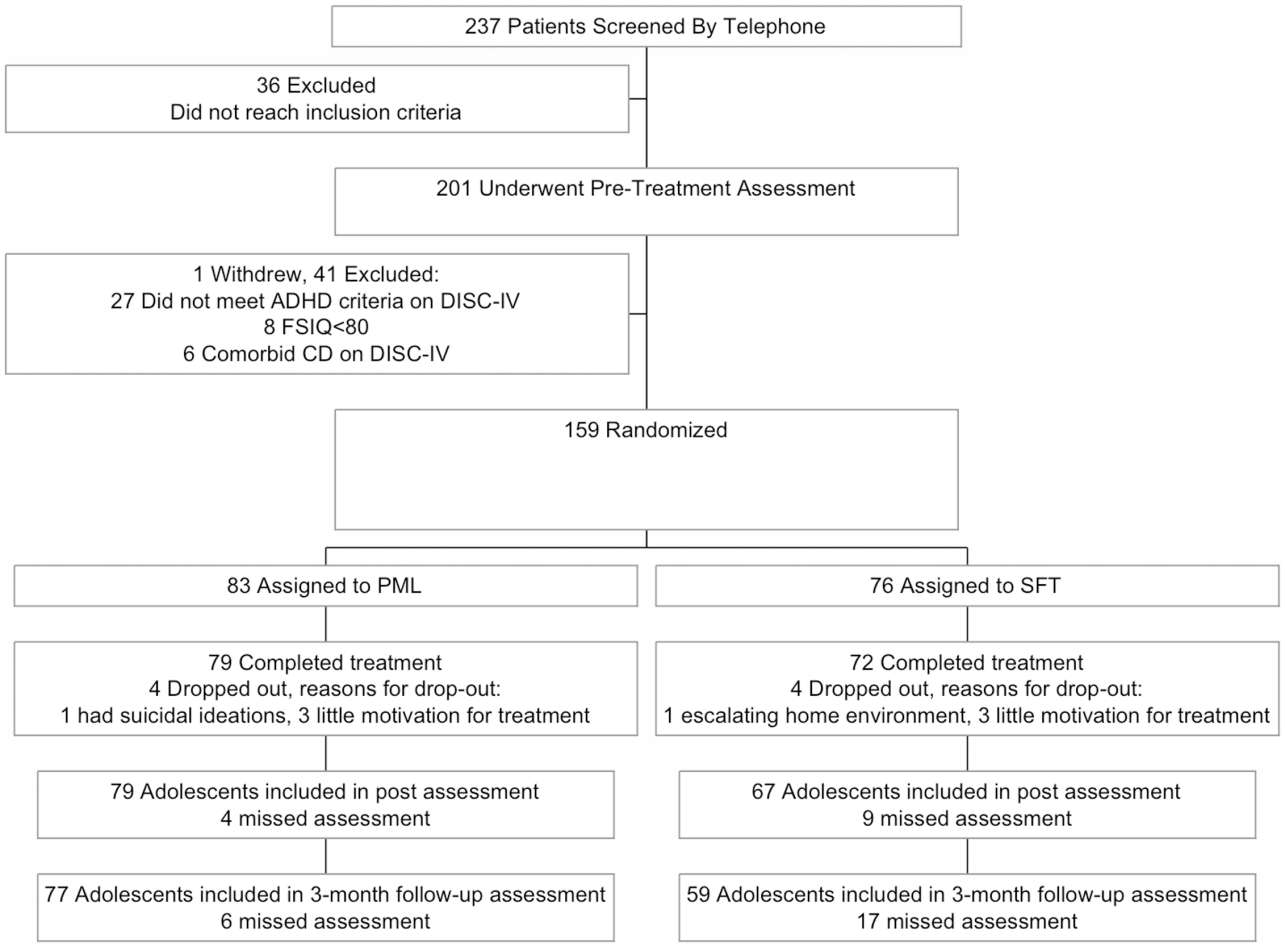
CONSORT flowchart.

Adolescents applied for the study in one of sixteen participating mental health care centers. Written informed consent was obtained from adolescents as well as their parents. After the pretest assessment, adolescents were randomly assigned to either PML (*N* = 83) or SFT (*N* = 76), using covariate adaptive randomization [40] by a blinded and independent researcher, and were stratified on gender and medication use (yes/no). To control for therapist effects, the 56 participating therapists (who all at least had a master degree in psychology) provided both treatments. Attrition in both treatments was low (PML *N* = 4, SFT *N* = 4) and the length of treatment was comparable across treatments (MPML = 9.3 weeks, SDPML = 2.6 MSFT = 9.1 weeks, SDSFT = 2.9). Adherence was high in both treatments and no treatment contamination was found. Posttest took place within a week after treatment and follow-up test approximately three months after treatment. All assessments and treatments took place in the same outpatient mental health care center where the participant applied for treatment and were conducted by blinded research assistants. Parent-rated questionnaires were completed by the primary caregiver, usually the mother. The Ethics Committee of the University of Amsterdam approved this study (2010-KP-1079). Details of adolescent trial flow are presented in Fig 1.

### Instruments

#### Treatments

*Similarities*: In our RCT we compared two treatments: PML and SFT [10]. Both are individual treatments that consist of 8 adolescent sessions of 45–60 minutes and 2 parental sessions. In both treatments the adolescent and therapist work together using a workbook and treatment manual. In both treatments motivational interviewing plays an important role: the attitude of the therapist as well as wording in the workbook are based on motivational interviewing. For example, the adolescents choose their own treatment goals (not those of their parents or teachers), which were written down and were always visible during treatment. Also, assignments were not presented as “homework”, but were formulated as an experiment for the upcoming week. In that way, the adolescent was free to choose a treatment strategy that fitted his/her life and the strategy could be adjusted according to their experiences. For a more detailed description of both treatments, see Boyer et al [10].

*Plan My Life*: (PML [15]) is a CBT in which in every session a fixed subject is discussed (e.g. using a daily planner or a to do list). For each subject possible planning- and organization strategies are presented for which the adolescent can compose their own strategy. The chosen strategy is than posited as an experiment that he/she will try the following week. Whenever needed, negative thoughts about the new strategy are challenged and helping thoughts formulated. All session strategies that have been tried the past week are discussed, including successes, possible room for improvement, and associated cognitions.

In the *Solution Focused Treatment* (SFT [16]) every session the adolescent discusses a problem he/she encounters. Following fixed questions, the adolescent is guided towards a solution for the posited problem. The fixed questions are: 1) What is the subject you chose? Describe the situation, 2) How is the situation, as it is now, a problem for you?, 3) How would you like it to be?, 4) What are solutions you used in the past and what are other possible solutions to the problem?, 5) Does the situation, as it is now, have advantages?, 6) Would you like to change the situation now/later/not at all, 7) If you choose to change, what is your plan? If you choose to change later or not at all, what are your considerations (pros and cons)? In this treatment the therapist does not tell the adolescent or parent what to do and does not teach them a new skill, but guides them towards a solution using the fixed questions.

*Differences between treatments*: The important difference between both treatments lies in the content. Whereas in PML every week a fixed subject is discussed regarding planning and organizing and planning skills are actively learned, in SFT the adolescent/parent has to choose the subjects themselves and is guided to his/her own solution.

#### Outcome measures

*ADHD symptoms* of the adolescents are measured using the Disruptive Behavior Disorder rating scale parent version (DBD [41] [42]). The DBD contains four scales composed of the DSM-IV criteria for ADHD Inattention, ADHD Hyperactivity/Impulsivity, Oppositional Defiant Disorder, and Conduct Disorder. In children with ADHD factor scores for parent ADHD ratings were found to have high internal consistency and adequate test-retest reliability [43]. In this study the ADHD symptoms scale is calculated summarizing the ADHD Inattention and the ADHD Hyperactivity/Impulsivity scales (18 items, scores range from 0 to 54). Analyses are conducted on raw scores, with a higher score indicating more symptoms of ADHD.

*Planning problems* of the adolescent are rated by parents using the Dutch translation of the Behavior Rating Inventory of Executive Function (BRIEF [44] [45]): A normative behavioral rating scale for children 5 to 18 years old, designed to elicit everyday planning behavior as observed by the parents in natural everyday environments. The BRIEF has appropriate psychometric properties including internal consistency ranging from .80 to .98 and test-retest reliability ranging from .76 to .88 [46]. The Plan/Organize scale measures the child’s capacity to anticipate future events, set goals, develop appropriate steps to carry out associated tasks or actions, and manage current and future-oriented task demands (12 items, scores range from 12 to 36). Analyses are conducted on raw scores, with higher scores indicating more planning problems.

Outcome measures are assessed at pretest one week before treatment, at posttest within a week after treatment and follow-up test approximately three months after treatment. The DBD was completed by 93.7% of the primary caregivers before treatment, 82.4% at posttest, and 67.3% at follow-up. The BRIEF was completed by 93.7% of the primary caregivers before treatment, 82.4% at posttest, and 66.7% at follow-up. To investigate our primary research question “Are qualitative treatment–subgroup interactions present in the data of our RCT?” analyses were conducted on the difference score between pretest and three-month follow-up on both the DBD and the BRIEF questionnaire. To answer our secondary research question: “Do the two treatment alternatives have a different effect on the trajectories of therapeutic change of ADHD-symptoms and planning problems for different subgroups of adolescents?”, we analyzed the difference between pre- and posttest on both the DBD and the BRIEF and the difference between post- and follow-up test on both the DBD and the BRIEF. The effect found between pre- and posttest is considered to be the short-term effect of treatment. The effect between post- and three-month follow-up test is considered to reflect the treatment maintenance.

#### Patient Characteristics

In total, 12 patient characteristics were assessed as potential moderators involved in qualitative treatment-subgroup interactions. Demographic variables included were gender, age, and medication use. Age was evaluated as a continuous variable while gender (male/female) and medication use (yes/no) were treated as categorical variables. Also, the average education level of both parents was assessed on a 4-point Likert-scale, with 1 representing the lowest educational level and 4 the highest. Parental education was treated ordinal. Several additional baseline adolescent characteristics were also included in the analyses as moderators:

*Full Scale IQ* was measured by two subtests of the Dutch Wechsler Intelligence Scale for Children (WISC-III-NL [38]) that correlate highly with the Total IQ: Vocabulary and Block Design (FSIQ [39]). The variable full scale IQ was treated as a continuous variable.

*ADHD-symptoms* of the adolescent were measured at pretest using the DBD rating scale parent version [41] [42]. The DBD contains four scales composed of the DSM-IV criteria for ADHD Inattention, ADHD Hyperactivity/Impulsivity, Oppositional Defiant Disorder, and Conduct Disorder (for psychometrics see outcome measure ADHD-symptoms). The ADHD symptoms scale is calculated, summarizing the ADHD Inattention and the ADHD Hyperactivity/Impulsivity scales, with a higher score indicating more symptoms of ADHD and was thus treated as a continuous variable.

*ADHD subtype* was assessed by the DISC-IV, a structured diagnostic interview, that establishes ADHD group membership and subtype status based on a diagnostic algorithm, including a check of cross-situational impairment [37]. The DISC-IV has shown substantial reliability (kappa = .79) in diagnosis of ADHD by parent-report [37]. Based on the DISC-IV the adolescents were classified into the inattentive subtype, the hyperactive/impulsive subtype and the combined subtype. This variable was treated as a categorical variable.

*Planning problems* of the adolescent were rated by parents at pretest using the BRIEF [44] [45]. This is a normative behavioral rating scale for children 5 to 18 years old, designed to elicit everyday EF as observed by the parents in natural everyday environments (for psychometrics see outcome measure ADHD-symptoms). In this study the Plan/Organize was used: higher scores on this subscale indicates more planning problems, and was therefore treated as a continuous variable.

*Depressive symptoms* were measured using the self-reported Child Depression Inventory (CDI [47] [48]). The CDI has demonstrated high internal consistency of .94 for normal subjects and .80 for psychiatric populations [49]. Scores range from 0 to 54, in which higher scores indicate more depressive symptoms, and was therefore treated as a continuous variable.

*Anxiety symptoms* were evaluated using the Screen for Child Anxiety Related Emotional Disorders (SCARED [50] [51]). The SCARED demonstrated good internal consistency (a = .74 to .93), test-retest reliability (intraclass correlation coefficients = .70 to .90) and discriminative validity (both between anxiety and other disorders and within anxiety disorders) [50]. Adolescents filled in 69 items, which added up to a total anxiety score, ranging from 0 to 138: Higher scores indicate more anxiety symptoms, and was thus treated as a continuous variable.

*ODD/CD symptoms* (DBD [41] [42]) were measured with the 24 items of the ODD and CD scales of the parent rated DBD (for psychometrics see outcome measure ADHD-symptoms). Scores range from 0 to 72, in which higher scores indicate more externalizing symptoms, and was therefore treated as a continuous variable.

*General Impairment* was measured using the Impairment Rating Scale (IRS [52]). Parents answered six questions on a scale from 0 to 10, resulting in a total score ranging from 0 to 60, with higher scores indicating high general impairment, and was thus treated as a continuous variable. The IRS demonstrated acceptable to excellent stability over time, evidence of concurrent, convergent, and discriminant validity, and appeared highly effective for discriminating between children with and without ADHD [53].

In the analyses, raw scores were used. Table 1 shows the adolescents’ scores on all variables: Independent t-tests and chi-squared tests showed no group differences on all patient characteristics between both treatments.

**Table 1:** Pre-treatment adolescent characteristics.

	All (*N* = 159)		PML (*N* = 83)		SFT (*N* = 76)		
	*M/N*	*SD*/%	*M/N*	*SD*/%	*M/N*	*SD*/%	Comparison
**Gender (*N* boys)**	117	73.6	63	75.9	54	71.1	*χ*^*2*^(1) = .48, *ϕ* = .06
**Age in years**	14.4	1.2	14.4	1.2	14.4	1.3	*t*(157) = .19, *d =* .00
**FSIQ**	103.4	11.8	102.5	11.6	104.3	11.9	*t*(157) = -.97, *d =* .15
**Parental education**^a^	3.1	0.8	3.2	0.7	3.1	0.8	*t*(146) = .55, *d* = .13
***N* Medication**	124	78.0	62	74.7	62	81.6	*χ*^*2*^(1) = 1.09, *ϕ* = .08
**ADHD Subtype**							*χ*^*2*^(2) = 5.06, *ϕ* = .18
ADHD-Inattentive	112	70.4	62	74.7	50	65.8	
ADHD-Hyperactive/impulsive	8	5.0	6	7.2	2	2.6	
ADHD-Combined	39	24.5	15	18.1	24	31.6	
**Other measures**							
ADHD symptoms	25.3	9.3	25.6	9.5	25.0	9.2	*t*(157) = .40, *d =* .06
Planning problems	28.2	4.4	28.0	4.5	28.4	4.4	*t*(157) = -.54, *d =* .09
ODD/CD symptoms	6.6	5.1	6.8	5.3	6.5	5.0	*t*(157) = .37, *d =* .06
Depression symptoms	10.0	6.1	10.1	6.5	10.0	5.7	*t*(157) = .13, *d =* .02
Anxiety symptoms	25.3	9.3	27.0	19.4	24.1	18.3	*t*(157) = .99, *d =* .15
Overall Impairment	31.5	11.5	32.3	11.7	30.6	11.3	*t*(157) = .93, *d =* .15

*Note*: ADHD, Attention Deficit Hyperactivity Disorder; CD, Conduct Disorder; FSIQ, Full Scale IQ; ODD, Oppositional Defiant Disorder; PML, Plan My Life; SFT, Solution Focused Treatment.

^a^ On this variable data are missing. Cramer’s *ϕ* effect size: .10 is small, .30 is medium, .50 is large; Cohen’s *d* effect size: 0.2 is small, 0.5 medium, 0.8 large.

### Statistical analyses

Intent-to-treat analyses were conducted. Missing parent and adolescent data were imputed using stochastic regression [54]. To address our research questions, we subjected the data to an analysis with the recently developed method QUINT [18]. We will first elaborate on the conceptual basis of QUINT and later on in this section explain how we set out the analysis of our data using this method. As mentioned before, the goal of QUINT is to find the best partition of the total group of adolescents with ADHD, based on their pretreatment characteristics, into two or three mutually exclusive types of subgroups that are characterized as follows: In the first subgroup type, (℘_1_), the adolescents assigned to treatment PML would show a clearly better outcome than the adolescents assigned to SFT; in the second subgroup type, (℘_2_), the reverse is true; in the third (optional) subgroup type, (℘_3_), the adolescents assigned to PML would show more or less the same outcome as the adolescents assigned to SFT. The subgroups may comprise one or several types of adolescents as defined by different (combinations of) dichotomized pre-treatment characteristics (moderators).

QUINT is looking for an optimal partition of the total group of adolescents so that the qualitative treatment-subgroup interaction that is related to that partition has the largest possible practical significance. To achieve this, two conditions with regard to the subgroups ℘_1_ and ℘_2_ need to be satisfied: (a) In both subgroups the difference in outcome between PML and SFT should be substantial, and (b) each of the two subgroups should comprise a sufficient number of adolescents. QUINT uses a weighted compound criterion that implies that these two conditions are optimized simultaneously. The difference in outcome between the PML and SFT treatment conditions included in condition (a) can be formalized in terms of either a difference in treatment means or a treatment effect size (Cohen’s *d* [55]).

**Fig 2:**
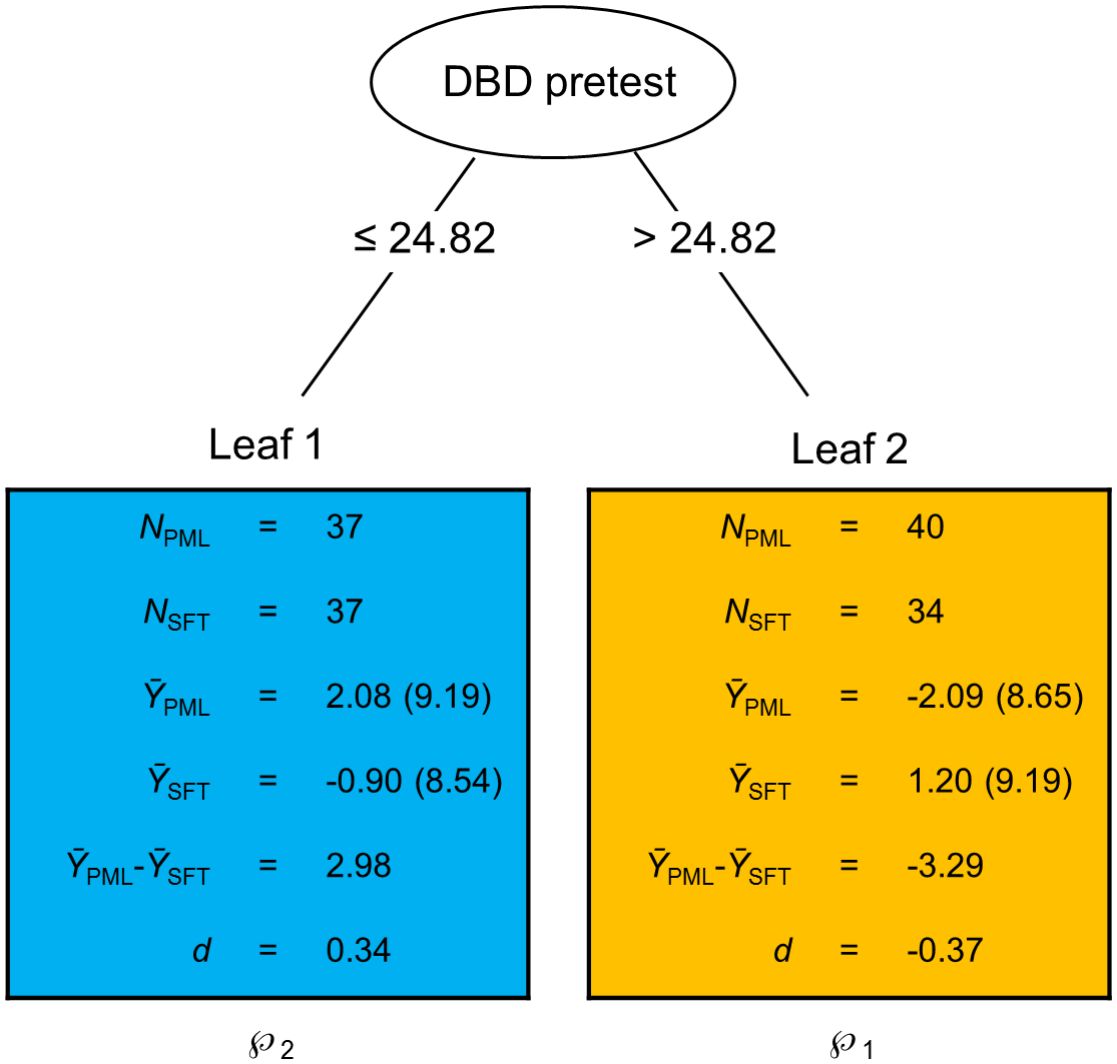
Result of the application of QUINT on posttest-to-follow-up difference in parent-rated ADHD symptoms (DBD; Disruptive Behavior Disorder rating scale parent version). Smaller outcome values are preferred as these refer to more reduction of parent-rated ADHD symptoms. The ellipsis in the figure represents the root node, which corresponds to the complete group of clients. This ellipsis contains the split variable, with below it the corresponding split point. The rectangles represent the leaves of the tree, that is, the final subgroups of adolescents; each rectangle contains the sample size of the corresponding subgroup, the outcome means (and standard deviations) for the plan my life and solution-focused treatment conditions (*Ȳ*_PML_ and *Ȳ*_SFT_), the uncorrected difference in means (*Ȳ*_PML_−*Ȳ*_SFT_), and the uncorrected corresponding effect size *d*. Assignment of the leaves to the partition classes is represented by ℘_1_ and ℘_2_.

To optimize this criterion, QUINT uses a stepwise tree-building algorithm. We refer to King and Resick [56] for a conceptual introduction to tree methods in psychological treatment research, and note that QUINT is an innovative member of this family of methods that is custom-made to identify qualitative treatment-subgroup interactions. It should also be noted that QUINT, like related nonparametric methods, makes no assumptions about the probability distribution of the assessed variables. The QUINT algorithm sequentially splits the total group of adolescents into subgroups, while optimizing in each step the weighted compound criterion mentioned above, with the resulting series of splits being represented by a tree structure like Fig 2 (which we will further discuss in the Results section). Starting with the total group of adolescents in the so-called root node, each background characteristic is considered as a candidate splitting variable to divide this group into two child nodes [24]. For each of the candidate splitting variables, all possible split points and corresponding assignments of the child nodes to subgroups ℘_1_ and ℘_2_ are evaluated; subsequently, the split point and assignment of the child nodes are chosen that maximize the QUINT criterion. Lastly, across all candidate splitting variables, the variable (along with its maximizing split point and assignment of child nodes to the subgroups) is selected that attains the highest value of the QUINT criterion. After this first split, the stepwise binary splitting procedure is continued. In each step, all end nodes (leaves) of the current tree then become candidate parent nodes. For each candidate parent node, the split (i.e., splitting variable, split point, and assignment of all end nodes or leaves of the tree to subgroups ℘_1_, ℘_2_ and ℘_3_) is selected that maximizes the QUINT criterion. The QUINT criterion values are subsequently compared across all candidate parent nodes and the node which implies the highest criterion value then is subdivided according to its optimal split. Note that from the second split on, leaves may be assigned to ℘_1_, ℘_2_ and ℘_3_ (instead of to ℘_1_ and ℘_2_ only as after the first split), and that after each split all leaves are allowed to be re-assigned to the three subgroups.

The QUINT procedure uses three types of criteria to stop the tree building process: Firstly, after the first split it tests the presence of a qualitative interaction on the basis of a so-called qualitative interaction condition, which reads that in each of the two leaves, the absolute value of the treatment effect size exceeds a critical minimum effect size value (*d*_min_). If QUINT does detect a qualitative treatment-subgroup interaction, the first split will be executed and the stepwise binary splitting procedure is continued. Secondly, the algorithm stops if no longer a split can be found that implies a higher criterion value than in the previous step. Thirdly, QUINT takes into account some additional stopping criteria including a maximum value for the number of leaves, and the fact that each leaf should contain a minimum number of adolescents assigned to PML and SFT.

The tree growing procedure may result in a large tree with a high criterion value for the data at hand, which may not be replicable with future data. QUINT controls for this so-called overfitting by a pruning procedure, which implies that the maximal tree is pruned back to an optimal subtree. This procedure yields a sequence of nested subtrees, for which the number of leaves (*L*) can be considered a complexity parameter. For each of these subtrees, QUINT has determined a criterion value. Yet, these values are positively biased since they have been determined on the basis of the same data as the ones that were used to build the tree. To overcome this problem, QUINT relies on a bootstrap procedure that yields an estimation of the biases, which further allows calculation of bias-corrected criterion values. The final tree selected by QUINT then is the one with the highest bias-corrected criterion value. Otherwise, QUINT also allows for a bootstrap-based bias correction procedure for the differential treatment effect sizes in the leaves of the finally selected tree, which may give insight into the generalizability of the QUINT solution. As the bootstrap samples have the same sample size as the data, the bootstrap-based bias correction procedure automatically takes into account possible small sample sizes of the data at hand. For a formalization and detailed description of the above, as well as for reports on the results of an extensive simulation study to address the problem of inferential errors (along with a number of strategies and recommendations to control for this), we refer to Dusseldorp and Van Mechelen [18]. To analyze RCTs with QUINT, the R-package quint has been developed by Dusseldorp, Van Mechelen and Doove [57]. In this package some freedom is left to the user (e.g., regarding parameters related to the qualitative interaction condition and tree complexity [58]). In the current version, clients with one or more missing values on any of the variables are omitted from the analysis (so-called listwise deletion). Therefore, if desired, missing data should be imputed before the QUINT analysis. The R-package quint can be freely downloaded from the Comprehensive R Archive Network (CRAN) and can handle continuous and dichotomous background characteristics. For the analyses in the present paper, we used a slightly extended version of this package that can also handle polytomous categorical background characteristics. This extension can be obtained from the authors.

In this study, the QUINT analyses were performed using the default effect size criterion. When using this criterion, Dusseldorp and Van Mechelen [18] propose to set the weight for the first constituent of the criterion equal to 1/log(1+ *d*_max_), where *d*_max_ is the realistic maximum for treatment effect size. Based on a recent review and meta-analyses on psychological interventions for ADHD [11] [59] [60], we set *d*_max_ equal to 1. As value for the weight of the second constituent of the optimization criterion, Dusseldorp and Van Mechelen [18] propose 1/log(0.50*N*), where *N* is the sample size. For reliable estimation of the mean outcome in a treatment group, we set the minimum number of adolescents assigned to PML and SFT in each leaf equal to 20. We used the default values for the critical minimum value in the qualitative interaction criterion for the absolute value of the standardized mean difference in treatment outcome (*d*_min_ = 0.30, based on results of a simulation study by Dusseldorp and Van Mechelen [18]), and the maximum number of leaves. Lastly, we set the number of bootstrap samples in the pruning procedure equal to 200, which is a relatively large number leading to more stable results than the default of 25 bootstrap samples. Note that we set out the analysis of our data based on simulation results of Dusseldorp and Van Mechelen (2014) for data sets with a number of potential treatment moderators ranging from 5 to 20 and a sample size equal or larger than 200. In this simulation study the impact of the number of moderators on Type I and Type II error rates appeared to be negligible. That being said, we cannot exclude that in the case of a sample size smaller than 200, there may be an effect of the number of potential treatment moderators on the risk of inferential errors.

## Results

### ADHD symptoms

To answer the first research question, “Are qualitative treatment–subgroup interactions present in the difference scores between pretest and three-month follow-up?”, we analyzed the difference scores for parent-rated ADHD (DBD). The test of the qualitative interaction condition after the first split (i.e., QUINT’s first stopping criterion, see Statistical analyses section) revealed that no qualitative treatment-subgroup interaction is present in the data. This result implies that there is no need for personalized treatment allocation for adolescents with ADHD when it comes to ADHD symptoms.

Next, we addressed for parent-rated ADHD the second research question on qualitative treatment-subgroup interactions in the two segments of the trajectory of therapeutic change from pretest to follow-up, namely from pretest to posttest, and from posttest to follow-up. With regard to the short-term effect of treatment, that is, the change in ADHD symptoms from pre- to posttest, QUINT again revealed no qualitative treatment-subgroup interaction. However, regarding the treatment maintenance (in other words, the change in ADHD-symptoms from post- to three-month follow-up test), QUINT *did* reveal a qualitative treatment-subgroup interaction in the data. The analysis resulted in a tree with two leaves (shown in Fig 2). These results show that adolescents with less ADHD symptoms (DBD pretest score ≤ 24.82), should preferably receive SFT over PML (Fig 2, Leaf 1); indeed, for these adolescents, improvement on ADHD symptoms between post- and follow-up test was on average 2.98 points higher when assigned to SFT compared to PML (*d* = 0.34). On the other hand, for adolescents with more ADHD symptoms at baseline (DBD pretest score > 24.82) greater treatment gains were observed when assigned to PML compared to SFT (Fig 2, Leaf 2). For these adolescents, improvement on ADHD symptoms was on average 3.29 points higher when assigned to PML compared to SFT (*d* = -0.37). After applying the bias-correction procedure to the two leaves, we found that the improvement on ADHD symptoms for the adolescents in Leaf 1 was on average 0.54 points higher when assigned to SFT compared to PML (*d* = 0.05), and that the improvement on ADHD symptoms for the adolescents in Leaf 2 was on average 1.17 points higher when assigned to PML compared to SFT (*d* = -0.11). The values of these bias-corrected effect sizes imply that the detected qualitative treatment-subgroup interaction is small.

**Fig 3:**
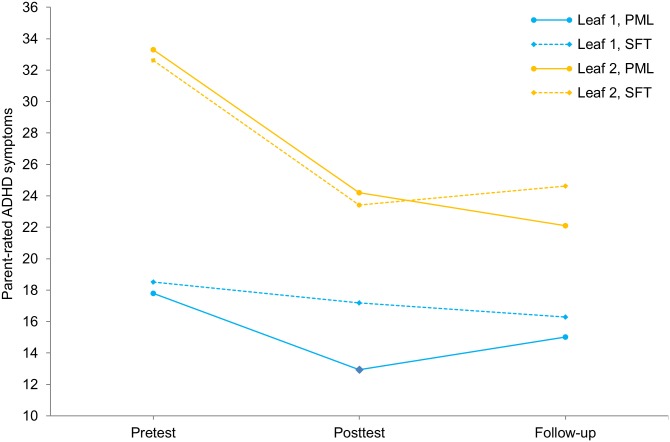
Change in parent-rated ADHD symptoms (DBD) from pretest to follow-up up for the two subgroups resulting from the application of QUINT on posttest-to-follow-up difference in parent-rated ADHD symptoms.The y-axis represents the raw scores on parent-rated ADHD symptoms. Leaf 1 is represented in blue, and Leaf 2 in orange. The plan my life (PML) and solution-focused treatment (SFT) groups within each leaf are represented by solid lines and dashed lines, respectively.

In order to more clearly understand the subgroup-specific trajectories across all three assessments, we examined the change in the mean outcome values in the two leaves across the entire time span from pretest to follow-up. Fig 3 shows this change in ADHD symptoms for the induced subgroups of adolescents, while further drawing a distinction between the two treatment conditions. The figure suggests that whereas from posttest to follow-up the adolescents in Leaf 1 benefit relatively more from SFT and the adolescents in Leaf 2 benefit relatively more from PML, this does neither apply to the time from pretest to posttest, nor for the entire time span from pretest to follow-up. From pretest to posttest the subgroups rather seem to be involved in a *quantitative* treatment-subgroup interaction (with PML outperforming SFT in both subgroups, but with the advantage of PML being larger for the adolescents in Leaf 1). This interaction, however, appeared not to be significant in a post-hoc factorial analysis of variance on the difference score between pre- and posttest (with a first factor pertaining to treatment and a second one to the leaves, *F*(1,144) = 1.84, *p* = .177, ω_p_^2^ = .006). Furthermore, the relatively larger advantage of PML for the adolescents in Leaf 1 in the change in symptoms from pretest to posttest, is undone by the qualitative treatment-subgroup interaction in the change in symptoms from posttest to follow-up (leading to a non-significant treatment-leaf interaction with regard to the change in symptoms from pretest to follow-up, *F*(1,144) = 1.03, *p* = .311, ω_p_^2^ = .000).

### Planning problems

With regard to the first research question “Are qualitative treatment–subgroup interactions present in the difference scores between pretest and three-month follow-up?”, we investigated these scores for parent-rated planning problems (BRIEF). QUINT revealed that no qualitative treatment-subgroup interaction is present in the data. This implies that there is no need for personalized treatment allocation for adolescents with ADHD when it comes to planning problems.

**Fig 4:**
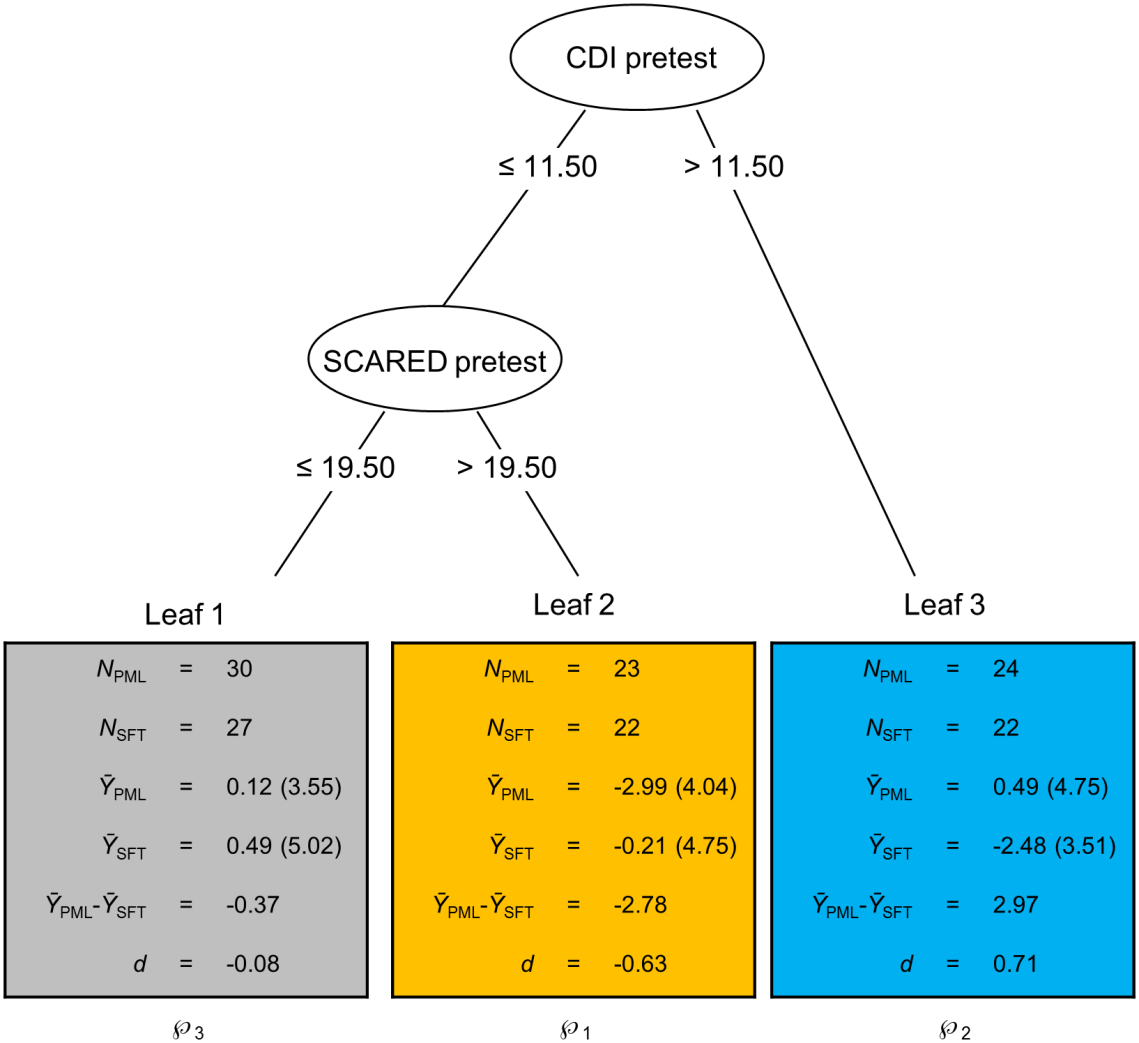
Result of the application of QUINT on posttest-to-follow-up difference in parent-rated planning problems (BRIEF). Smaller outcome values are preferred as these refer to more reduction of parent-rated planning problems. The ellipses in the figure represent the internal nodes containing the split variables (CDI, Child Depression Inventory; SCARED, Screen for Child Anxiety Related Emotional Disorders), with below each ellipsis the corresponding split point. The upper ellipsis represents the root node corresponding to the complete group of clients. The rectangles represent the leaves of the tree, that is, the final subgroups of adolescents; the quantities contained in the rectangles are analogous to those in Fig 2. Assignment of the leaves to the partition classes is represented by ℘_1_, ℘_2_ and ℘_3_.

For the second research question on qualitative treatment-subgroup interactions in the two segments of the trajectory of therapeutic change from pretest to follow-up, difference scores between pre-and posttest, and between posttest and follow-up were subjected to QUINT analyses. With regard to the difference scores between pre- and posttest, QUINT revealed that no qualitative treatment-subgroup interaction is present, whereas for the difference scores between posttest and follow-up a qualitative interaction *is* present in the data. Regarding the latter, QUINT constructed a tree with three leaves. The pruning procedure indicated that this was also the optimal tree size. The structure of the tree is shown in Fig 4. From this figure it appears that for adolescents in Leaf 3 with more depressive symptoms at baseline (CDI pretest score 11.5) greater treatment gains were observed when assigned to SFT compared to PML: For these adolescents, improvement on planning problems was on average 2.97 points higher when assigned to SFT compared to PML (*d* = 0.71). On the other hand, adolescents in Leaf 2 with less depressive symptoms (CDI pretest score ≤ 11.5), and more anxiety symptoms (SCARED pretest score > 19.50) improvement on planning problems was on average 2.78 points higher when assigned to PML compared to SFT (*d* = -0.63). Finally, for adolescents in Leaf 1 with low depression and low anxiety levels, effects of both treatments are comparable. After applying the bias-correction procedure to the two leaves with the most extreme differential treatment effects, we found that the improvement on planning problems for the adolescents in Leaf 2 was on average 1.30 points higher when assigned to PML compared to SFT (*d* = -0.27), and that the improvement on planning problems for the adolescents in Leaf 3 was on average 1.73 points higher when assigned to SFT compared to PML (*d* = 0.36). These bias-corrected values imply that the effect size of the detected qualitative treatment-subgroup interaction is between small and medium. Post-hoc analyses revealed that the three subgroups do not differ from each other on other pre-treatment characteristics besides depressive and anxiety symptoms.

**Fig 5:**
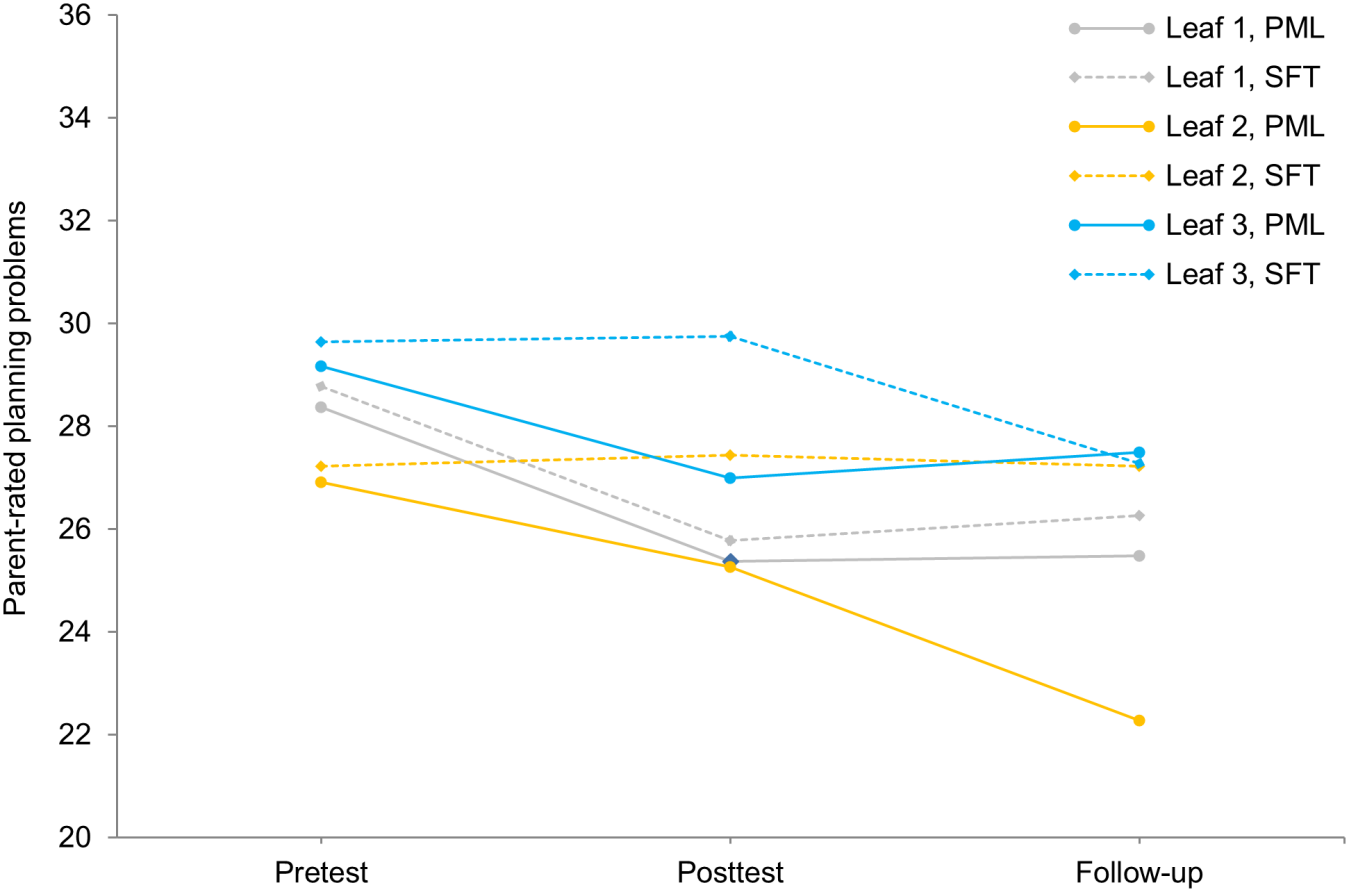
Change in parent-rated planning problems from pretest to follow-up for the three subgroups resulting from the application of QUINT on posttest-to-follow-up difference in parent-rated planning problems (BRIEF). Leaf 1 is represented in grey, Leaf 2 in orange, and Leaf 3 in blue. The plan my life (PML) and solution-focused treatment (SFT) groups within each leaf are represented by solid lines and dashed lines, respectively.

In order to more clearly understand the subgroup-specific trajectories across all three assessments, we again examined the change in the mean outcome values in the three leaves across the entire time span. This change in symptoms is shown in Fig 5 for the parent-rated BRIEF for the three different subgroups, while further drawing a distinction between the two treatment conditions. Whereas from posttest to follow-up, the three subgroups are involved in a qualitative treatment-subgroup interaction, this is the case neither for the change in symptoms from pretest to posttest nor for the change in symptoms from pretest to follow-up. Rather, based on Fig 5, the three subgroups seem to be involved in a *quantitative* treatment-subgroup interaction with regard to the change in symptoms from pretest to posttest, with PML outperforming SFT in leaves 2 and 3, and with no between-treatment difference in Leaf 1. In a post-hoc ANOVA, however, this interaction appeared not be significant, *F*(2,142) = 0.99, *p* = .375, ω_p_^2^ = .000. With regard to the change in symptoms from pretest to follow-up, Fig 5 also suggests the presence of a quantitative treatment-subgroup interaction with PML outperforming SFT in Leaf 2, and with no between-treatment difference in leaves 1 and 3. In a post-hoc ANOVA, this interaction appeared to be significant, indeed, *F*(2,142) = 4.18, *p* = .017, ω_p_^2^ = .041. Moreover, a post-hoc Tukey test confirmed that PML significantly outperformed SFT in Leaf 2 whereas the treatment groups did not significantly different from each other in Leaves 1 and 3. Importantly, this implies that adolescents with less depressive symptoms (CDI pretest score ≤ 11.5 and more anxiety symptoms (SCARED pretest score > 19.5) should preferably receive PML, while for other adolescents the effects of PML and SFT are comparable.

## Discussion

This was the first study to explore moderators involved in qualitative treatment-subgroup interactions in adolescents with ADHD, using an innovative statistical technique (QUINT). Our results show that no qualitative treatment–subgroup interactions were present in the data of our RCT for the change in symtoms from pretest to three months after treatment, thereby answering our primary research question. This implies that, when comparing the two CBTs under study in adolescents with ADHD (one that aimed on enhancing planning problems and one that did not), there is no need for personalized treatment allocation when focusing on improvement of ADHD symptoms and planning problems as outcome variables. As noted above, Boyer et al. [10] reported for the same RCT a significant improvement in ADHD- and comorbid symptoms and general impairment of the adolescents with large effect sizes, but only marginal differences in effect between treatments. This lack of treatment differences is therefore not due to qualitative treatment-subgroup interactions.

Our second research question focused on the trajectories of therapeutic change for different subgroups of adolescents in both treatments, with a distinction between short-term effects of treatment and maintenance effects up to three months after treatment. Our results showed no qualitative treatment–subgroup interactions for the short-term effect of treatment. However, with regard to the treatment maintenance from posttest to follow-up, qualitative treatment-subgroup interactions were found: In terms of improvement in parent-rated planning problems, adolescents with more comorbid anxiety symptoms but with low depressive symptoms fared better with PML, while adolescents with high depressive symptoms showed better results with SFT. Yet, when taking the change in symptoms of the identified subgroups into consideration from pretest to three-month follow-up, only the results in favor of PML were upheld. In addition, in terms of improvement in parent-rated ADHD symptoms, effects of PML were more positive for adolescents with more severe ADHD, while SFT showed more positive effects for adolescents with less symptoms of ADHD. Nevertheless, these results had small effect sizes only and these subgroups were not upheld in the course from pretest to follow-up. The most robust results were therefore, that adolescents with more anxiety symptoms but low levels of depression fared better with PML than with SFT with regard to improvement of parent-rated planning problems.

QUINT provided specific cut-off scores for treatment allocation (CDI: cut-off point = 11.50; SCARED: cut-off point = 19.50). But as this study is exploratory, and found subgroups should be replicated in a follow-up RCT, for now these cut-off scores should not be implemented in clinical practice. If these cut-off scores are replicated they could augment the clinical relevance of this study enabling treatment assignment. Nevertheless, as other factors not included in this study like treatment preference, could also influence treatment effectiveness, treatment allocation should not be based solely on cut-off scores [61]. What is also important to note, is that the subgroups generated by QUINT that are based on these cut-off scores, do not represent the normal or clinical range of scores on these questionnaires. In other words, the interpretation of these scores depends on the norms of the corresponding questionnaire. For example, effects of PML were more positive than SFT for adolescents with a score on the CDI below 11.50, but a higher score than 19.50 on the SCARED. Depending on the age and gender of the participant, a score of 19.50 on the SCARED can be considered a low to average score as compared to the norm [51]. The cut-off point of the CDI can be considered average [48]. All in all, this means that for adolescents with low to average depression, and with some to high anxiety (but not necessarily a clinical anxiety level), PML is the treatment of preference.

Our findings regarding the moderating role of comorbid anxiety symptoms on treatment outcome, are in line with results of previous studies. For example, a non-randomized study on the effects of CBT in adolescents with ADHD that amongst other elements included training of planning and organizing skills [62], also showed that a subgroup of adolescents with comorbid anxiety improved more in comparison to adolescents with ADHD only [63]. Moreover, in the MTA-study the subgroup of children with comorbid anxiety showed more improvement when receiving behavioral treatment [25] [26] [27] than those without comorbid anxiety. This might indicate a subgroup within ADHD that is particularly, or at least differently, sensitive to effects of (cognitive) behavioral treatment. The MTA Cooperative Group suggested that, as anxiety reduces after treatment of ADHD, at least some anxiety in individuals with ADHD might be attributed to the stress of ADHD-related problems [27]. This is consistent with the idea that anxiety, in individuals with ADHD, is more strongly associated with negative affectivity and disruptive behavior than with fearfulness or phobic symptoms [26]. If this would be the case, in our study one would expect anxiety not to be a moderator, because in our RCT two CBTs were compared and after both ADHD as well as anxiety had improved [10]. Because the more anxious group of adolescents with ADHD showed more positive results when receiving a CBT focusing on planning skills than when receiving a CBT without such an aim, this suggests that the anxiety experienced by adolescents with ADHD could be attributed to poorer executive functions (e.g., [64] [65] [66]). This is supported by research showing increased working memory deficits and increased rates of sluggish cognitive tempo in children with ADHD and comorbid anxiety [67]. However, this mediation hypothesis needs further testing.

An alternative explanation for the superior effects of PML as compared to SFT in the subgroup with low depression but higher anxiety (see Leaf 2, Figs 4 and 5), could be the change of use of medication for ADHD. Keep in mind that adolescents could not change medication status (yes/no) or dose during treatment, but could only change these after posttest. When testing this hypothesis exploratory, we found an (albeit nonsignificant) trend that in this specific subgroup, adolescents receiving PML increased their dose of methylphenidate after posttest (M = 11.12, SD = 21.46), while adolescents receiving SFT decreased their dose (M = -1.69, SD = 13.88), t(24) = 1.72, *p* = 0.08 (note that due to missing values on medication dose at pretest and follow-up, this test is based on a subset of the adolescents in Leaf 2). While this is the case for this specific subgroup, this treatment difference is not found for the whole group of adolescents in this study. This may imply that adolescents with low depression and higher anxiety that received PML have become more motivated to adhere to medication as compared to SFT. But this finding may also imply that the increases in methylphenidate were responsible for the treatment gains of adolescents receiving PML. However this hypothesis needs further testing.

Further, a notable result is that pretreatment characteristics may influence the effects of treatment differently during different phases of treatment. In this study the pretest to follow-up change in symptoms of the found qualitative subgroups, clearly shows that the PML and SFT treatment alternatives have a different effect on the change in planning problems during treatment and during treatment maintenance for different subgroups of adolescents. This supports the idea of two clearly separate treatments; both follow different trajectories that seem to converge to comparable endpoints after three months. Moreover, these results show that it is important to consider the phase of treatment when investigating moderator-effects: in this study differential effects were found in different phases of treatment. This may imply that different characteristics determine the effect of treatment during different phases. To our knowledge, previous studies on moderation of treatment effects in individuals with ADHD, have focused primarily on effects of treatment between pre- and posttest and may therefore miss potential moderators [25] [26] [27] [28] [29] [30] [31] [32]. We would therefore advise to take the phase of treatment into account when interpreting the effects of moderators and to also investigate moderators of follow-up treatment effects in future studies.

This study has several limitations. First, in this study several adolescent characteristics but no family characteristics, such as parenting self-efficacy, were included as potential moderators. Second, because all adolescents were included in a treatment condition, their parents who completed ratings scales (i.e. outcome measures) were potentially biased by expectancy effects. Therefore, in future studies more objective tests (i.e. blinded measures) assessing ADHD core symptoms are needed. Also, due to the lack of an adequate control-group in our RCT like a waitlist or a treatment as usual group effectiveness of both treatments could not be proven [10]. Third, as in all analyses without a priori hypotheses, there is no guarantee that for all results that are generated using QUINT, a clinically satisfactory interpretation can be provided. Fourth, a broad concern related to the goal of finding subgroups involved in treatment-subgroup interactions pertains to the risk of inferential errors. On the one hand, these include Type II errors, which reflect a possible lack of power to detect true interactions (e.g., [68]). In general, a reliable detection of interactions requires larger samples than a reliable detection of main effects [69], and perhaps considerably larger than those enrolled in traditional clinical trials in the field of behavior therapy research. On the other hand, one should also beware of Type I errors, that is, erroneous claims about the occurrence of apparent interactions that cannot be replicated in follow-up studies [70] [68] [71] [72]. Tree-based methods are especially vulnerable at this point as they rely on a very large search space based on a very huge number of covariate split-point combinations and therefore need large sample sizes [18]. Even though this study is the largest RCT in adolescents with ADHD to date, for QUINT to show replicable results, a larger sample size would be preferred. To be sure, we made use of a bias-corrected bootstrap procedure to prevent ourselves from overfitting the data at hand. However, Dusseldorp and Van Mechelen [18] state that the safest way to deal with QUINT, especially in case of applications with relatively smaller sample sizes, as an exploratory tool to generate meaningful hypotheses. Further, these hypotheses should be tested in follow-up confirmatory research with new RCTs that make use of a stratified sampling scheme in which the strata are constructed on the basis of the splitting variables and split points as identified by QUINT [18]. As a final note, we would like to emphasize that the fact that QUINT (as all post-hoc methods) brings with it the risk of inferential errors does not preclude that in trials like the one studied in this paper the use of subgroup analyses such as QUINT can be meaningful. Conducting an RCT requires lots of time, money and effort, which is mainly an argument for getting as much information as possible out of the data. Exploratory analyses such as the one reported in this paper are still of great value. However, it is of utmost importance that results are regarded as tentative until they can be replicated.

Taken together, this study has several clinical implications. Our RCT showed improvements with large effect sizes for both PML and SFT, but without between-treatment differences on most measures (except for parent-rated planning problems [10]). The present study supplements these findings by showing that there were no significant moderators of qualitative treatment-subgroup interactions of therapeutic change from pretest to three months after treatment: Both treatments harvest comparable results. There was however a *quantitative* interaction present in the data, that showed that for adolescents with ADHD with comorbid anxiety symptoms in combination with low levels of comorbid depression, PML appears to be the treatment of preference. Of additional clinical relevance are the cut-off points generated by QUINT, with which clinicians can directly allocate adolescents to the most effective treatment. All in all, personalized treatment assignment appears unnecessary, as the effects of two CBTs are comparable for all adolescents with ADHD, except for a subgroup of adolescents for whom PML is the treatment of preference.

## Supporting Information

S1 CONSORT Checklist CONSORT checklist. (DOC)

S1 Protocol Original trial study protocol. (DOC)

S2 Protocol Translated trial study protocol. (DOCX)

S3 Protocol Original Ethics Protocol. (PDF)

S4 Protocol Translated Ethics Protocol. (PDF)
