# Supplementary material for: Qualitative Treatment-Subgroup Interactions in a Randomized Clinical Trial of Treatments for Adolescents with ADHD: Exploring What Cognitive-Behavioral Treatment Works for Whom
Source: PLoS One. 2016 Mar 15;11(3):e0150698. doi: 10.1371/journal.pone.0150698 (PMC4792426; doi:10.1371/journal.pone.0150698)
Supplement: S1 Protocol — (DOC) [file pone.0150698.s002.doc]

**Beschrijving onderzoek:**

In dit onderzoek wordt de effectiviteit onderzocht van twee verschillende behandelprotocollen voor adolescenten met ADHD (zie ook CE-nummer 2009-KP-1026). Van het eerste protocol, de behandeling van planning- en organisatievaardigheden door middel van cognitieve gedragstherapie, wordt verwacht dat met name een effect te zien zal zijn bij adolescenten waarbij executieve functiestoornissen op de voorgrond staan. Van het tweede protocol, een steunende en structurerende behandeling, wordt verwacht dat een effect te zien zal zijn bij adolescenten waarbij stemmingsklachten op de voorgrond staan. Het betreft een gerandomiseerd groepsexperiment met twee condities (de twee behandelprotocollen) met een voormeting, een nameting en twee folluw-up metingen: 3 maanden na behandeling en 1 jaar na behandeling. In totaal zal getracht worden 250 adolescenten bij dit multi-centre onderzoek te betrekken. Gedurende beide behandelingen kan medicatiegebruik voortgezet worden, mits de dosering tijdens behandeling niet gevarieerd wordt. Beide behandelingen zullen bestaan uit 9 sessies bij gesuperviseerde psychologen.

**Overzicht vragenlijsten/taken onderzoek:**

**Inclusiecriteria**

Jongeren in de leeftijd van 12 tot en met 16 jaar, die naar de middelbare school gaan en een diagnose ADHD hebben (gecombineerde subtype of aandachtsgestoorde subtype). De ADHD symptomen zullen worden gemeten met de ouderversie van het Diagnostisch Interview Schema voor Kinderen (DISC-IV; Ferdinand e.a., 1998) en de Vragenlijst voor Gedragsproblemen bij Kinderen (VVGK; Oosterlaan, Bayens, Scheres, Antrop, Roeyers, & Sergeant, 2008). Daarnaast hebben de jongeren een geschat totaal IQ van 80 of hoger op de WISC-III-R en is er sprake van significante plannings- en organisatieproblemen, gemeten met de Behavioral Rating Inventory of Executive Functioning (BRIEF; Smidts & Huizinga, 2009; Goia, Isquith, Guy, & Kenworthy, 2000).

- **Diagnostic Interview Schedule for Children (DISC)**

Het gestructureerde interview (Ferdinand & Van der Ende, 2002) wordt bij de primaire opvoeder afgenomen door de onderzoeksmedewerker. Hiervoor worden alleen de (relevante) secties ADHD, ODD en CD afgenomen.

- **Wechsler Intelligence Scale for Children (WISC-III-R)**

Een verkorte versie van de WISC-III-R wordt afgenomen bij de jongeren (2 subtests: Woordenschat en Blokpatronen), op basis waarvan een schatting van het IQ wordt gemaakt.

- De **Vragenlijst voor Gedragsproblemen bij Kinderen (VvGK)**, ouder- en leerkrachtversies (Oosterlaan e.a., 2008). Deze vragenlijst meet ADHD-kenmerken, en kenmerken van oppositioneel opstandig gedrag en antisociale gedragsproblemen.
- De vertaalde versie van de **Behavioral Rating Inventory of Executive Functioning** (BRIEF; Smidts & Huizinga, 2009) wordt gebruikt om het executief functioneren te bepalen (Gioa e.a., 2000). De lijst – 86 items - is bedoeld voor kinderen tussen de 5 en 18 jaar en wordt door de ouders en de leerkrachten van de jongeren ingevuld. Items hebben betrekking op moeilijkheden met executieve functies als werkgeheugen, emotieregulatie, planning, inhibitie en cognitieve flexibiliteit. Er is een Nederlandse vertaling beschikbaar en er zijn normgegevens van zich normaal ontwikkelende kinderen.

**Exclusiecriteria**

Wanneer blijkt uit het interview met de ouders dat er sprake is van comorbide verslavingsproblematiek, stemmingsproblemen met gevaar voor suicide, overheersende gedragsproblemen of heftig escalerende thuissituaties, worden jongeren uitgesloten van deelname.

**Metingen**

**Psychopathologie**

- De Child Behavior Checklist zal worden ingevuld door de ouders en de Youth Self Report door de jongeren zelf (CBCL en YSR; Achenbach, 1991), om een beeld te krijgen van de gedrags- en stemmingsproblemen van de jongeren.

**Uitkomstmetingen (voor de voor- en nameting)**

- **Ecologische variabelen**

1. De jongeren zullen aan het begin van de training aangeven welke 5 gedragingen zij zouden willen verbeteren. Dit doelgedrag zal iedere week als onderdeel van de training gemeten worden aan de hand van een 5-punts likertschaal.
2. De ouders zullen aan het begin van de behandeling gevraagd worden welk gedrag van hun kind zij verbeterd zouden willen zien. Dit doelgedrag zal iedere week per mail gemeten worden aan de hand van een 5-punts likertschaal.

- **Ouder-adolescent conflict**

Om de mate van conflict tussen ouders en adolescent te inventariseren wordt gebruik gemaakt van de vertaalde versie van de Conflict Behavior Questionnaire (CBQ-20 verkorte versie; Prinz, 1977).

- **Schoolprestaties –en functioneren**

1. De schoolprestaties zullen worden bijgehouden door het monitoren van de cijfers van de jongeren op school.
2. De jongeren zelf zullen de School Vragenlijst (SVL; Vorst & Smits, 2007) invullen. Deze vragenlijst geeft informatie over hoe een leerling school ervaart: de motivatie voor schoolvakken, de tevredenheid met school en het zelfvertrouwen waarme schooltaken uitgevoerd worden. De test is Nederlands genormeerd, voor 9- tot 16 jarige kinderen en heeft een goede betrouwbaarheid en validiteit (Cotan, 2008).

- **Executief Functioneren**

1. De vertaalde versie van de Behavioral Rating Inventory of Executive Functioning (BRIEF; Smidts & Huizinga, 2009) wordt gebruikt om het executief functioneren te bepalen (Gioa e.a., 2000). De lijst – 86 items - is bedoeld voor kinderen tussen de 5 en 18 jaar en wordt door de ouders en de leerkrachten van de jongeren ingevuld. Items hebben betrekking op moeilijkheden met executieve functies als werkgeheugen, emotieregulatie, planning, inhibitie en cognitieve flexibiliteit. Er is een Nederlandse vertaling beschikbaar en er zijn normgegevens van zich normaal ontwikkelende kinderen.

- **Executieve Functie Taken**

De in de behandeling getrainde planning en organisatievermogens worden gemeten met de volgende standaard neuropsychologische taken:

- 1. Met de dierentuin plattegrond taak en de sleutelzoektaak van de Behavioral Assessment of the Dysexecutive Syndrome (BADS-NL; Tjeenk-Kalff & Krabbendam, 2007) worden de ecologische planning en organisatie van de jongeren in kaart gebracht. Deze tests zijn geschikt voor de doelgroep en er zijn Nederlandse normen beschikbaar.
  2. Met de Tower Test uit de Delis-Kaplan Executive Function System (D-KEFS; Delis, Kaplan, & Kramer, 2001) zal eveneens de planning van de jongeren in kaart worden gebracht.
  3. Met de Trail Making Test (Delis, Kaplan, & Kramer, 2007) worden de planning en flexibiliteit van de jongeren getest. Deze test heeft verschillende parallelversies, waardoor deze goed bruikbaar is bij voor- en nametingen. Er zijn Amerikaanse normen beschikbaar.
- **Predictorvariabelen**
  1. De flankertaak die in dit onderzoek gebruikt zal worden is een variant op de flankertaak van Eriksen, een taak die binnen de cognitieve psychologie veelvuldig wordt toegepast om te onderzoeken hoe de mensen informatie die afleidt negeren. De jongeren krijgen in deze taak een reeks van 5 figuren op het scherm te zien met de instructie om zo snel mogelijk de knop in te drukken die hoort bij het middelste figuur. Op twee van de vier figuren moet met de rechter knop worden gereageerd, op de andere twee figuren met de linker knop. De helft van de reeksen zijn congruent, dat wil zeggen dat de response behorende bij het middelste figuur (de ‘target’) overeen komt met die van de andere 4 figuren (de ‘flankers’). De andere helft van deze reeksen, de incongruente reeksen, bevatten een target die correspondeert met de andere knop. In de variant die bij dit onderzoek gebruikt wordt zal een conditie worden toegevoegd waarbij de jongeren niet beloond worden voor hun prestatie op de taak en een conditie waarbij de jongeren wel beloond zullen worden voor hun prestatie op de taak in de vorm van een klein cadeau.
  2. De bis/bas schalen (Colder & O’Connell, 2004; Nederlandse vertaling Luman, 2007) zullen door ouders worden ingevuld om de mate van gevoeligheid van de jongeren voor beloning en straf te meten.

**Na pilot studie en interactie met subsidiegever veranderd:**

1. toegevoegd Child Depression Inventory (CDI, zelf rapportage), Screen for Child Anxiety Related Emotional Disorders (SCARED, zelf rapportage), Homework Problems Checklist (20 items ouder rapportage) en de Classroom Performance Survey (23 items, leerkracht rapportage) Impairment Rating Scale (ouder/leerkracht rapportage),
2. verwijderd YSR (Youth Self Report, Achenbach, 1991)
