# Supplementary material for: Qualitative Treatment-Subgroup Interactions in a Randomized Clinical Trial of Treatments for Adolescents with ADHD: Exploring What Cognitive-Behavioral Treatment Works for Whom
Source: PLoS One. 2016 Mar 15;11(3):e0150698. doi: 10.1371/journal.pone.0150698 (PMC4792426; doi:10.1371/journal.pone.0150698)
Supplement: S2 Protocol — (DOCX) [file pone.0150698.s003.docx]

**Description research:**

This study evaluates the effectiveness of two treatment manuals for adolescents with ADHD (see also CE-number 2009-KP-1026). From the first manual, the treatment of planning and organization skills through cognitive behavior therapy is expected that one will see an effect with adolescents with executive functioning difficulties. For the second one, a supportive structuring solution focused protocol, is expected that this will be of effects for those with internalizing difficulties. The study is a randomized group experiment with two conditions (the manuals) with a pre, post, follow-up 3 months and 1 year after treatment. It will be attempted to recruit 250 adolescents in this multicenter trial. During treatment medication treatment is available but must not be varied in dose/type. Both treatments have 9 sessions with supervised psychologists.

**Measures:**

**Inclusion**

Adolescents in the age of 12-16 years, in secondary school and a diagnosis of ADHD (combined or inattentive subtype). ADHD symptoms are assessed with the parent version of the Diagnostic Interview Schedule for Children (DISC-IV; Ferdinand e.a., 1998) and the Disruptive Behavior Disorders Rating Scale; Dutch version Vragenlijst voor Gedragsproblemen bij Kinderen [VVGK; Oosterlaan, Baeyens, Scheres, Antrop, Roeyers, & Sergeant, 2008]. Next to that an estimated IQ of 80 or above on the WISC-III-R and significant planning problems on the Behavioral Rating Inventory of Executive Functioning (BRIEF; Smidts & Huizinga, 2009; Goia, Isquith, Guy, & Kenworthy, 2000).

- **Diagnostic Interview Schedule for Children (DISC)**

Structured interview (Ferdinand & Van der Ende, 2002) with primary caretaker by research assistant, only relevant sections ADHD, ODD and CD are assessed.

- **Wechsler Intelligence Scale for Children (WISC-III-R)**

Short version WISC-III-R (2 subtests: Vocabulary and Block Design), based on this an estimation of IQ

The **Vragenlijst voor Gedragsproblemen bij Kinderen (VvGK)/ Disruptive Behavior Disorders Rating Scale**, parent and teacher version (Oosterlaan e.a., 2008). This questionnaire assessed ADHD, ODD and CD symptoms.

- The translated version of the **Behavioral Rating Inventory of Executive Functioning** (BRIEF; Smidts & Huizinga, 2009) of executive functions (Gioa e.a., 2000). This questionnaire– 86 items – is for children between 5 and 18 years. Items related to difficulties in executive functions, working memory, emotion regulation, planning, inhibition, and cognitive flexibility. Dutch translation and norms are available.

**Exclusion**

Parent indicated comorbid addiction, mood disorders with risk of suicide, severe behavioral problems or crises-home situations.

**Measures**

**Psychopathology**

- De Child Behavior parents Youth Self Report adolescents (CBCL en YSR; Achenbach, 1991), behavior/mood.

**Outcomes (pre-post)**

- **Ecological variables**

1. 5-goal behavior 5-point likert scale (adolescent).
2. 5-goal behavior 5-point likert scale (parent).

- **Parent-adolescent conflict**

Conflict Behavior Questionnaire (CBQ-20 short version; Prinz, 1977).

- **Schoolachievement–en functioning**

1. Grades.
2. School Vragenlijst (SVL; Vorst & Smits, 2007/ School Attitude Questionnaire). Dutch norms for 9- to 16 year olds adequate reliability/validity (Cotan, 2008).

- **Executive Functioning**

1. Translated version of the Behavioral Rating Inventory of Executive Functioning (BRIEF; Smidts & Huizinga, 2009/ Gioa e.a., 2000).

- **Executive Functioning Tasks**

Neuropsychological measures:

- 1. Zoo map task and Key search of the Behavioral Assessment of the Dysexecutive Syndrome (BADS-NL; Tjeenk-Kalff & Krabbendam, 2007).
  2. Tower Test from the Delis-Kaplan Executive Function System (D-KEFS; Delis, Kaplan, & Kramer, 2001).
  3. Trail Making Test (Delis, Kaplan, & Kramer, 2007) planning flexibility of adolescents.
- **Predictorvariabels**
  1. flanker task (flanker task of Eriksen), task assessing interference inhibition.
  2. Parent rated bis/bas scales (Colder & O’Connell, 2004; Dutch translation Luman, 2007)

**After pilot study and interaction with grant committee changed:**

1. Added Child Depression Inventory (CDI, selfreport), Screen for Child Anxiety Related Emotional Disorders (SCARED, selfreport), Homework Problems Checklist (20 items parent report) and the Classroom Performance Survey (23 items, teacher report) Impairment Rating Scale (teacher and parent report),
2. Removed YSR (Youth Self Report, Achenbach, 1991)
