# Supplementary material for: Qualitative Treatment-Subgroup Interactions in a Randomized Clinical Trial of Treatments for Adolescents with ADHD: Exploring What Cognitive-Behavioral Treatment Works for Whom
Source: PLoS One. 2016 Mar 15;11(3):e0150698. doi: 10.1371/journal.pone.0150698 (PMC4792426; doi:10.1371/journal.pone.0150698)
Supplement: S3 Protocol — (PDF) [file pone.0150698.s004.pdf]

Algemeen deel

Project title

behandeling van adolescenten met ADHD

Responsible researcher (if a PhD-project is concerned list the professor involved)

Saskia van der Oord, Hilde Geurts, Pier Prins

Who conducts the research? (PhD-students, students, etc.)

Bianca Boyer

Responsible Department or Section

Clinical Psychology

Research location

Verschillende GGZ instelingen in Noord-Holland, Utrecht en Flevoland

Brief project description (max. 200 words)

In dit onderzoek wordt de effectiviteit onderzocht van twee verschillende kortdurende behandelprotocollen voor adolescenten met ADHD (zie ook het pilot onderzoek onder CE-nummer 2009-KP-1026). Adolescenten van 13 tot 16 jaar worden at random verdeeld over twee behandelingen. Van de eerste behandeling van planning- en organisatievaardigheden door middel van cognitieve gedragstherapie, wordt verwacht dat met name een effect te zien zal zijn bij adolescenten waarbij executieve functiestoornissen op de voorgrond staan. Van de tweede behandeling, een steunende en structurerende behandeling, wordt verwacht dat een effect te zien zal zijn bij adolescenten waarbij stemmingsklachten op de voorgrond staan. Effect van behandeling wordt gemeten voor behandeling, na behandeling en 3 maanden en 1 jaar na behandeling. In totaal zal getracht worden 250 adolescenten bij dit multi-centre onderzoek te betrekken. Gedurende beide behandelingen kan medicatiegebruik voortgezet worden, mits de dosering tijdens behandeling niet gevarieerd wordt.

Expected duration of the project

3 jaar

Expected number of participants

250

This project is comparable with the following submitted project(number)

2009-CP-1026

Inhoudelijk deel

A1. When classifying the research as Medical vs. Non-medical, does it comply with A1, meaning it can be listed under category D (see also Appendix 1, 2.4)?

Yes, it falls into category D

A2. Are consenting adults selected, as described in A2?

No, describe participant details and recruitment procedure
Please submit the information letter and the consent form as attachment

Comment:

Er worden adolescenten (12-16 jaar) geselecteerd met een diagnose ADHD. De adolescenten zullen worden geworven bij de deelnemende GGZ-instellingen op advies van de behandelend hulpverlener of door middel van informatiefolders en via de website www.adhdtrainingen.nl

A3. Are participants free to decide to participate and to stop for whatever reason, as listed under A3?

Yes

A4. Are participants subjected to a screening procedure to reduce the risks for adverse effects, as listed under A4?

No

Comment:

Er worden geen nadelige effecten van de behandeling verwacht voor de adolescenten of hun ouders

A5. Is there a risk for chance incidents that should be reported to the participant, as listed under A5?

No, the method precludes chance incidents

A6. Are participants fully informed before participating, and do they sign a consent form, as listed under A6?

Yes, please submit the information letter and the consent form as attachment

A7. Is participant privacy and anonymity guaranteed, as listed under A7?

Yes

A8. In case of deception, does the procedure comply with the conditions listed under A8? (full disclosure concerning risks, accurate debriefing)?

There is no deception

A9. Is there a risk that a substantial number of participants will drop out because the research is considered to be discomforting, as listed under A9?

No

B1. Does the research **fully** comply with the guidelines for Standard Research?

Yes, specify below and upload a concise research description (max 1 A4)

Answer:

B4. Psychometrie
