# Supplementary material for: Qualitative Treatment-Subgroup Interactions in a Randomized Clinical Trial of Treatments for Adolescents with ADHD: Exploring What Cognitive-Behavioral Treatment Works for Whom
Source: PLoS One. 2016 Mar 15;11(3):e0150698. doi: 10.1371/journal.pone.0150698 (PMC4792426; doi:10.1371/journal.pone.0150698)
Supplement: S4 Protocol — (PDF) [file pone.0150698.s005.pdf]

Translation Ethics Committee number 2010-CP-1079

Treatment of adolescents with ADHD

General information

Project Title

Treatment of adolescents with ADHD

Responsible researcher (if a PhD-project is concerned list the professor involved)

Saskia van der oord, Hilde Geurts, Pier Prins

Who conducts the research? (PhD-students, students, etc.)

Bianca Boyer

Responsible Department or section

Clinical Psychology

Research location

A range of Menthal Health Care institutes in provinces North-Holland, Utrecht and Flevoland

Brief Project Description (max. 200 words)

In this study the effectiveness of two short-term treatment protocols for adolescents with ADHD was investigated (also see the pilot study, Ethics Committee number 2009-KP-1026). Adolescents aged 13 to 16 years will be randomly assigned to one of both treatments. Because the first treatment is a cognitive behavioral therapy (CBT) aiming on enhancement of planning and organization skills, it is expected that effects will be especially visible in adolescents that have executive dysfunctions. Because the second treatment is a supporting solution focused treatment, it is expected that treatment effects will be especially visible in adolescents who have mood disorders. Treatment effects will be assessed at pretest, posttest, three months after treatment and one year after treatment. In total, we will try to recruit 250 adolescents for this multi-center study, provided that dose of medication will not be altered during treatment.

Expected duration of the project

3 years

Expected number of participants

250

This project is comparable with the following submitted project(number)

2009-CP-1026

Contentual information

A1. When classifying the research as Medical vs. Non-medical, does it comply with A1, meaning it can be listed under category D (see also Appendix 1, 2.4)?

Yes, it fall into category D

A2. Are consenting adults selected, as described in A2?

No, describe participant details and recruitment procedure

Comment:

Adolescents (12 to 16 years) with an AHD-diagnosis will be selected. Adolescents will be recruited at the participating mental health care institutes, by recommendation by their therapist or with information flyers or by our website www.adhdtrainingen.nl.

A3. Are participants free to decide to participate and to stop for whatever reason, as listed under A3?

Yes

A4. Are participants subjected to a screening procedure to reduce the risks for adverse effects, as listed under A4?

No

Comment:

No adverse effects are expected for the adolescents or their parents.

A5. Is there a risk for chance incidents that should be reported to the participant, as listed under A5?

No, the method precludes chance incidents.

A6. Are participants fully informed before participating, and do they sign a consent form, as listed under A6?

Yes, please submit the information letter and the consent form as attachment

A7. Is participant privacy and anonymity guaranteed, as listed under A7?

Yes

A8. In case of deception, does the procedure comply with the conditions listed under A8? (full disclosure concerning risks, accurate debriefing)?

There is no deception

A9. Is there a risk that a substantial number of participants will drop out because the research is considered to be discomforting, as listed under A9?

No

B1. Does the research **fully** comply with the guidelines for Standard Research?

Yes, specify below and upload a concise research description (max. 1 A4)

Answer:

B4. Psychometrics
